# Altitude thresholds for hemoglobin and plasma volume regulation in adult residents of different altitudes and their impact on hemoglobin concentration

**DOI:** 10.14814/phy2.71108

**Published:** 2026-10-02

**Authors:** Edgar Cristancho‐Mejía, Andrea Catalina Trompetero‐Gonzalez, William Fernando Benavides‐Pinzón, Walter Franz‐Joachim Schmidt

**Affiliations:** ^1^ Departamento Biología, Facultad de Ciencias Universidad Nacional de Colombia Bogotá Colombia; ^2^ Departamento de Ciencias Fisiológicas, Facultad de Medicina Universidad Nacional de Colombia Bogotá Colombia; ^3^ Escuela de Medicina y Ciencias de la Salud Universidad del Rosario Bogotá Colombia; ^4^ Department of Sports Medicine and Sports Physiology University of Bayreuth Bayreuth Germany

**Keywords:** altitude‐threshold, Andean residents, CO‐rebreathing, erythropoiesis, hemoconcentration

## Abstract

Life at altitude is characterized by increased erythropoietic activity and hemoglobin concentration ([Hb]). The first aim of the project was to identify the altitudes at which significant increases in hemoglobin mass (Hbmass) and decreases in plasma volume (PV) occur among residents living at different altitudes. The second aim was to evaluate the extent to which Hbmass and PV contribute to changes in [Hb] at moderate altitudes. The study included 133 young men and 148 women from six Andean regions at altitudes ranging from 970 to 2600 m. Hbmass was determined using carbon monoxide rebreathing. PV was calculated using Hbmass, [Hb], and hematocrit. Hbmass increased up to 2600 m by 35.9 ± 11.6 g, while PV decreased by 208 ± 53 mL (both *p* < 0.01). The first significant increase in Hbmass occurred at 2180 m and the first significant decrease in PV occurred at 1730 m. At 2600 m, Hbmass and PV contributed equally to the 1.2 g/dL increase in [Hb], that is, 0.56 and 0.61 g/dL, respectively. The threshold for a decrease in PV in Andeans is ~450 m lower than the threshold for an increase in Hbmass. The contribution of hemoconcentration to the increase in [Hb] at moderate altitudes is equal to the contribution of erythropoiesis.

## 1 INTRODUCTION

It is well known that hemoglobin concentration (Hb) increases with altitude (Mairbäurl et al., 2023; Weil et al., 1968). The most obvious mechanism is increased erythropoiesis, which results in the formation of a larger hemoglobin mass (Hbmass) within a few days or weeks when ascending from a low altitude, and over the long term when residing permanently at a higher altitude. However, for residents living up to an altitude of approximately 1600 m, there is no measurable increase in erythrocyte production due to the flat upper part of the O_2_ binding curve. This means that the arterial oxygen content either does not decrease or decreases only insignificantly (Weil et al., 1968). Above this altitude, for example, at 2600 m, where the ambient PO_2_ is ~25% lower than at sea level, the absolute Hbmass increases by ~6% (Mancera‐Soto et al., 2022). At 3800 meters, where the PO_2_ is lowered by ~40%, the Hbmass increases by ~27% (Schmidt et al., 2022). Although approximately 5% of the world's population lives above 1500 m (Mairbäurl et al., 2023) and [Hb] is a common diagnostic tool whose threshold values for anemia have been adjusted for altitude (Gassmann et al., 2019; Mairbäurl et al., 2023), there is no systematic data on an altitude threshold above which erythropoiesis increases linearly or exponentially.

In addition to the amount of hemoglobin in the blood [Hb] is determined by the liquid phase of blood, that is, plasma volume (PV). For example, PV can decrease by up to 18% due to intense anaerobic exercise and increase by 10% within 48 h after long, intense endurance exercise (Schmidt, Brabant, et al., 1990). These changes result in significant alterations in [Hb] without any change in Hbmass. When ascending to higher altitudes, PV decreases, resulting in an increase in [Hb] by ~4% after 1 day and by 9% after 7 days at 2500 m, for example, (Beidleman et al., 2017). The reverse process occurs when people living at 2600 m (Cristancho et al., 2026) or 3600 m descend to lower elevations (Wachsmuth et al., 2013). However, there is still no data available on a possible altitude threshold above which a chronic reduction in PV occurs.

Changes in [Hb] immediately after a change in altitude are well known to be caused by short‐term variations in PV. However, it is unclear to what extent increased Hbmass and/or reduced PV determine [Hb] in people living chronically at high altitudes (Tremblay et al., 2025). Interestingly, data from Stembridge et al. (Stembridge et al., 2019) and Claydon et al. (2005) suggest that the lower [Hb] of Sherpas compared to Andean residents living at the same altitude is largely due to higher PV rather than lower Hbmass, which is commonly assumed (Beall, 2000). Therefore, when diagnosing anemia, for example, it is necessary to be aware of ethnic differences that may cause variations in the ratio of Hbmass to PV (Boulares et al., 2025).

The primary objective of the study was to identify potential altitude thresholds for changes in hemoglobin mass and plasma volume in people living at moderate altitudes in South America. The second objective was to quantify the contribution of hemoglobin mass and plasma volume to altitude‐related changes in hemoglobin concentration in a healthy, nonanemic population of the Andes.

## 2 MATERIALS AND METHODS

### 2.1 Subjects and study design

A total of 281 individuals (133 males and 148 females) participated in this cross‐sectional study. See Tables 1 and 2 for study locations and anthropometric data. The study was conducted in accordance with the Declaration of Helsinki and was approved by the Ethics Committee of the Faculty of Natural Sciences at the National University of Colombia in Bogotá on April 24, 2009. All participants were informed of the study's objectives and procedures and of their right to withdraw from the study at any time without providing a reason. Written consent was obtained from all participants.

**TABLE 1:** Characteristics of the study locations.

Altitude (m)	Ambient temperature (°C)		Ambient P_O2_ (mmHg)	Ambient pressure (mmHg)
	Day	Night		
970	29.5	18.3	142.8 ± 0.5	677.5 ± 2.3
1470	25.5	16.0	133.2 ± 0.3	636.5 ± 1.3
1730	24.5	14.0	130.9 ± 0.5	625.4 ± 2.6
1920	23.3	11.9	127.7 ± 0.2	610.1 ± 1.1
2180	21.3	12.3	124.5 ± 0.3	594.6 ± 1.3
2600	19.4	7.2	118.6 ± 0.3	567.7 ± 1.2

*Note*: Presented are the average annual temperatures for the respective elevations, as well as the average values for ambient pressure and PO_2_ on the days of measurement.

**TABLE 2:** Anthropometric data of the study participants.

Altitude (m)	*n* =	Age (years)	Height (cm)	Body mass (kg)	BMI	LBM (kg)
*Males*						
970	17	23.5 ± 4.2	173.8 ± 5.7	69.3 ± 10.5	22.9 ± 2.8	59.7 ± 7.6
1470	12	20.1 ± 3.3	171.3 ± 8.2	63.0 ± 8.2	20.1 ± 3.3	53.5 ± 3.8
1730	22	21.6 ± 3.6	172.1 ± 7.9	66.6 ± 13.6	21.6 ± 3.6	56.8 ± 7.8
1920	15	20.2 ± 2.9	168.0 ± 6.6	58.6 ± 6.1	20.8 ± 2.3	52.3 ± 4.3
2180	12	20.1 ± 2.1	169.1 ± 4.4	62.7 ± 7.8	21.9 ± 2.1	54.3 ± 4.2
2600	55	22.1 ± 3.1	170.9 ± 5.3	63.7 ± 8.2	21.8 ± 2.7	55.3 ± 5.4
ANOVA *p*<		0.001		0.05		0.01
*Females*						
970	28	21.7 ± 4.5	159.9 ± 4.4	56.0 ± 6.0	21.9 ± 2.3	43.7 ± 4.2
1470	11	20.8 ± 4.4	157.8 ± 5.4	57.7 ± 10.5	23.1 ± 3.2	44.5 ± 5.6
1730	22	22.7 ± 3.4	157.4 ± 4.8	52.6 ± 7.1	21.3 ± 2.9	41.7 ± 3.5
1920	18	18.9 ± 0.9	157.6 ± 6.5	51.4 ± 6.7	20.8 ± 2.9	40.7 ± 2.2
2180	21	19.8 ± 4.0	156.6 ± 5.4	57.4 ± 8.8	23.4 ± 3.5	43.4 ± 4.6
2600	48	23.3 ± 3.8	158.1 ± 4.7	56.2 ± 8.3	22.5 ± 3.1	43.2 ± 5.1
ANOVA *p* <		0.001				

*Note*: Presented are means ±SD.

Healthy, non‐smoking participants were recruited from five regions around Bogotá, Colombia (Central Eastern Andes, altitudes between 1470 and 2600 m) and Tuluá/Cali, Colombia (Western Andes, 970 m, see Table 1). The participants' ethnicity corresponded to the regional distribution and differed only slightly between the two regions (Central Eastern/Western Andes: African origin, 5.1%/14.0%; European origin, 58.9%/55.4%; Native American origin, 36.0%/30.5%) (Ossa et al., 2016). All participants were born and had lived at their respective altitudes for at least the last 2 years. None of them practiced endurance sports or participated in a physical training program.

Body fat mass was determined using quadripolar impedance (Omron BF508 Bioimpedance Analysis, Omron, Osaka, Japan). Participants were weighed, and their fat percentage and fat‐free mass were determined in triplicate.

### 2.2 Hemoglobin mass (Hbmass) and blood volumes

Hbmass was determined using the CO rebreathing method according to Schmidt and Prommer (Prommer & Schmidt, 2007; Schmidt & Prommer, 2005). Briefly, a small amount of CO was inhaled over 2 min using a specially designed spirometer (Blood tec GmbH, Bayreuth, Germany). At 970 m, the CO dose was 1.2 mL/kg of body weight for males and 0.8 mL/kg for females and was adjusted according to the drop in air pressure at each altitude. Capillary blood samples were taken from the earlobe before and 7 min after the start of inhalation and the COHb% was measured three times in each sample using an ABL 700 (Radiometer, Copenhagen, Denmark). Hbmass was calculated from the difference in COHb% before and after inhalation, taking into account the amount of CO diffused into myoglobin (for a detailed description of the method, see references (Mancera‐Soto et al., 2022; Prommer & Schmidt, 2007; Schmidt & Prommer, 2005)). Blood volume (BV), red cell volume (RCV), and plasma volume (PV) were calculated using Formulas (1), (2), (3).

(1) $$\mathrm{BV}=\text{Hbmass}\times 100/{\left[\mathrm{Hb}\right]}_{\mathrm{ven}}/\mathrm{cf}.$$

(2) $$\mathrm{RCV}=\text{Hbmass}\times {\mathrm{Hct}}_{\mathrm{ven}}/{\left[\mathrm{Hb}\right]}_{\mathrm{ven}}$$

(3) PV=BV−RCV

[Hb]_ven_ and Hct_ven_ = venous [Hb] and venous hematocrit, respectively; cf. = cell factor to account for differences between central and peripheral hematocrit (Fåhraeus effect). Two different values for cf. were used to calculate plasma volume: 1. A constant value of 0.91 (Chaplin Jr. et al., 1953); 2. A cf. value that varies with increasing altitude (i.e., from 0.910 at 970 m to 0.938 at 2600 m (Sanchez et al., 1970)).

### 2.3 Blood samples

After resting in a seated position for 15 min, 5 mL of blood was drawn from a cubital vein. The samples were heparinized, and [Hb] was determined in triplicate in both samples immediately using an ABL 700 (Radiometer, Copenhagen, Denmark). Hematocrit (Hct) was determined by microcentrifugation at 3500 g, and oxygen saturation (SpO_2_) was measured in capillary blood obtained from a hyperemic earlobe, also using an ABL 700.

### 2.4 Statistics

SPSS Statistics Version 25 (IBM Corp., Armonk, NY, USA) software was used for statistical analysis. To compare the mean values of the different altitudes, a two‐sided ANOVA with two factors between subjects, that is, sex and altitude, was applied. Student's unpaired *t*‐test was used for post hoc tests to check for differences between males and females. In addition to the two‐sided ANOVA, a one‐sided ANOVA (altitude only) was performed for males and females separately. When the ANOVA indicated significance, we conducted paired *t*‐tests to assess differences from baseline values. To minimize the risk of type I errors, we applied a correction for multiple comparisons (Benjamini & Hochberg, 1995).

Simple bivariate linear regression analyses were performed with [Hb], as well as Hbmass, PV, and BV related to LBM as the dependent variables and ambient partial pressure for oxygen as the independent variable.

We then performed univariate analyses of variance using the dependent variables Hbmass, PV, and [Hb] to investigate the effects of the factors of sex and altitude level, as well as of the covariate LBM, on these variables. The effect size of each factor was reported as a partial ƞ^2^ value, which expresses the percentage of total variance that is accounted for.

A multiple regression analysis was performed to quantify the possible associations between [Hb] and the factors sex, Hbmass, PV, and SpO_2_ (Table 5a).

A further multiple linear regression analysis was performed using the data presented in Table 4. This analysis estimated the effects of the altitude‐related mean differences in Hbmass and PV as independent variables on the differences in [Hb] at increasing altitudes as dependent variable (Table 5b).

## 3 RESULTS

The anthropometric characteristics of the different altitude groups were relatively homogeneous for both males and females (see Table 2). The only differences observed were in age, with maximum differences of 3.4 years for males and 4.4 years for females, and in LBM, with maximum group differences of 7.4 kg for males.

Oxygen saturation was lower by up to 2.2% (males) and 1.5% (females) with increasing altitude, which was significant for residents at 1920 m and above (Table 3). At 2600 meters, there was a significant sex difference in SpO_2_, with lower values observed in males (93.8% ± 1.4% vs. 94.6% ± 1.2%, *p* < 0.01).

**TABLE 3:** Hematological data.

	SpO_2_ %	[Hb] g/dL	Hct %	Hbmass g	RCV mL	PV mL	BV mL	Hbmass g/kg	RCV g/kg	PV g/kg	BV g/kg	Hbmass g/kgLBM	RCV mL/kgLBM	PV mL/kgLBM	BV mL/kgLBM
**Males**															
970 m	96.0 ±1.2	15.6 ±0.9	46.2 ±2.2	827 ±158	2343 ±449	3501 ±686	5845 ±1110	11.8 ±0.8	33.5 ±2.3	50.0 ±4.6	83.5 ±6.1	13.7 ±1.0	38.7 ±2.9	57.8 ±5.8	96.5 ±7.9
1470 m	95.4 ±0.9	15.9 ±0.7	47.5 ±2.9	797 ±97	2271 ±293	3264 ±545	5535 ±795	12.7 ±0.9	36.2 ±2.9	51.8 ±4.4	87.9 ±5.8	14.2 ±0.7	40.5 ±2.3	57.2 ±4.6	97.7 ±4.8
1730 m	95.7 ±0.9	15.8 ±0.8	47.3 ±2.4	771 ±118	2204 ±338	3170* ±506	5374 ±811	11.8 ±1.5	33.6 ±4.4	48.4 ±7.1	81.9 ±11.0	13.6 ±1.2	38.8 ±3.5	55.8 ±5.7	94.6 ±8.3
1920 m	95.0* ±1.1	16.6*** ±0.7	49.7*** ±1.8	753 ±80	2143 ±260	2849*** ±331	4992** ±561	12.9* ±1.0	36.6* ±3.2	48.8 ±4.8	85.4 ±7.2	14.4* ±0.9	41.0 ±2.9	54.6 ±5.3	95.5 ±7.4
2180 m	95.2 ±1.1	16.3* ±1.1	48.6* ±3.4	801 ±99	2287 ±282	3109* ±413	5396 ±623	12.8* ±1.1	36.6* ±3.1	49.7 ±4.1	88.3 ±5.2	14.7* ±1.2	42.1* ±3.4	57.1 ±5.1	99.2 ±6.3
2600 m	93.8*** ±1.4	16.6*** ±0.9	49.4*** ±2.5	797 ±94	2300 ±282	2993*** ±367	5293** ±584	12.6* ±1.2	36.4* ±3.7	47.5* ±5.6	83.9 ±8.0	14.5* ±1.3	41.7** ±4.0	54.3* ±5.8	96.1 ±8.3
ANOVA *p* <	0.001	0.001	0.001			0.001	0.05	0.01	0.01	0.1		0.05	0.01	0.1	
**Females**															
970 m	96.1 ±1.0	13.8 ±0.8	41.0 ±2.4	483 ±64	1411 ±194	2408 ±242	3820 ±404	8.8 ±0.9	25.6 ±2.7	43.8 ±4.1	69.4 ±6.0	11.2 ±1.1	32.7 ±3.3	55.9 ±4.6	88.6 ±6.8
1470 m	95.6 ±0.8	14.1 ±0.8	43.0* ±1.5	504 ±58	1512 ±196	2418 ±285	3930 ±467	8.9 ±1.3	26.7 ±4.1	42.7 ±6.4	69.4 ±10.2	11.5 ±1.6	34.5 ±5.4	54.9 ±7.7	89.4 ±12.9
1730 m	96.1 ±1.2	14.4* ±1.1	42.0 ±3.1	464 ±62	1351 ±182	2193* ±331	3544 ±470	8.9 ±0.9	25.8 ±2.7	41.9 ±5.7	67.7 ±7.3	11.2 ±1.2	32.4 ±3.5	53.6* ±6.3	85.0 ±8.4
1920 m	94.5** ±1.1	14.7*** ±0.8	44.6*** ±2.4	461 ±71	1397 ±227	2040** ±254	3438* ±452	9.0 ±1.1	27.3 ±3.6	40.0* ±5.3	67.3 ±8.3	11.3 ±1.3	34.2 ±4.1	50.1** ±4.8	84.3 ±8.0
2180 m	95.3* ±1.2	15.0*** ±0.9	44.4*** ±2.7	520 ±72	1517 ±209	2305 ±350	3821 ±526	9.1 ±0.9	26.6 ±2.6	40.6 ±6.4	67.2 ±8.6	12.0* ±1.0	34.9* ±2.9	53,2* ±6.8	88.1 ±8.9
2600 m	94.6** ±1.2	15.1*** ±0.8	44.7*** ±2.7	523* ±62	1551** ±190	2261* ±275	3812 ±421	9.4* ±1.1	27.8** ±3.1	40.6* ±5.0	68.5 ±7.3	12.2** ±1.2	36.1*** ±3.8	52.6* ±5.4	88.6 ±8.0
ANOVA *p* <	0.001	0.001	0.001	0.001	0.001	0.01	0.05	0.05	0.05	0.1		0.001	0.01	0.05	

*Note*: Presented are means ±SD. Significance of differences from 970 m: * = *p* < 0.05, ** = *p* < 0.01, *** = *p* < 0.001.

Abbreviations: BV = blood volume, Hbmass = hemoglobin mass, PV = plasma volume, RCV = red cell volume, SpO_2_ = peripheral oxygen saturation.

The hematological data, as well as the absolute and relative blood volumes, are presented in Table 3. On average, there was a linear relationship between ambient PO_2_ and [Hb], Hbmass, and PV (Figure 1a–c). [Hb] and Hct levels were higher in residents living at altitudes of 1920 m and above. The greatest differences were observed between 970 and 2600 m. (Δ[Hb]: 1.0 g/dL, ΔHct: 3.2% in males; 1.3 g/dL and 3.7% in females). Absolut Hbmass was not different between the groups of males, but it was highest in females living at 2600 m. Relative Hbmass (g/kg LBM) was higher in male and female residents living at an altitude of 2180 m or higher. The difference between 970 m and 2600 m was 0.8 g/kg LBM (5.8%) in males and 1.0 g/kg LBM (8.9%) in females. In both sexes, the absolute PV was significantly lower at altitudes of 1730 m and above. Lower values of relative PV (g/kg LBM) were observed at lower altitudes in females than in males. The difference in PV between the extreme altitudes was 3.4 mL/kg LBM (6.1%) for males and 3.3 mL/kg LBM (6.0%) for females. The effects of altitude on PV are much stronger when PV is calculated with the cf. according to Sanchez (Sanchez et al., 1970) (see Tables S1 and S2 in Supplementary Material). There were no differences in blood volume, either in absolute or relative terms.

**FIGURE 1:**
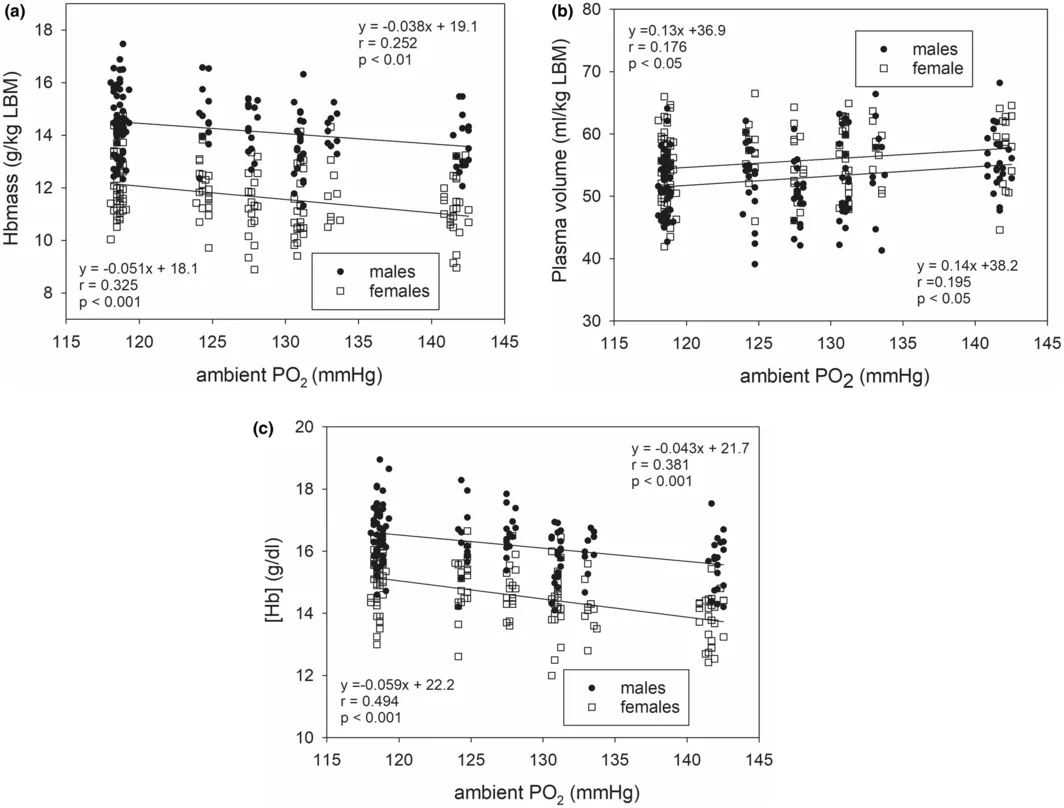
Relationship between ambient oxygen pressure and hemoglobin mass (Hbmass, a), plasma volume (PV, b), and hemoglobin concentration ([Hb], c). Statistical information for the male groups is presented in the upper corners, for females in the lower corners.

With the exception of LBM‐related PV, all hematological data presented in Table 3 showed highly significant differences between males and females.

Table 4 shows the absolute effects of sex, LBM per kilogram, and altitude level compared to the lowest altitude (970 m) on Hbmass, PV, and Hb. The analysis of variance revealed highly significant influences of LBM (*ƞ* ^2^ = 0.51) and sex (*ƞ* ^2^ = 0.34) on Hbmass as well as a positive influence of altitude (*ƞ* ^2^ = 0.082) which was significant for residents at 2180 m and above. The effect of sex on Hbmass is partly independent from LBM, as demonstrated by the significant difference of 2.52 ± 0.15 g/kg LBM (*p* < 0.001) between males and females.

**TABLE 4:** Effect of sex, altitude levels, and lean body mass (LBM) on hemoglobin mass (Hbmass), plasma volume (PV) and hemoglobin concentration ([Hb]).

	Sex	Altitude					LBM kg	Constant term
	Male	1470 m	1730 m	1920 m	2170 m	2600 m		
Hbmass (g)	138.0*** ±11.9	10.4 ±17.0	−7.6 ±13.4	14.3 ±14.7	36.2** ±13.9	35.9** ±11.6	11.9*** ±0.7	−32.0 ±33.7
PV (mL)	230** ±55	−79 ±78	−150* ±62	−257*** ±68	−121 ±64	−208*** ±53	47.4*** ±3.3	383* ±155
[Hb] (g/dL)	1.5 ±0.2	0.4 ±0.2	0.5* ±0.2	1.0*** ±0.2	1.1*** ±0.2	1.2*** ±0.2	__	13.7*** ±0.5

*Note*: The estimated effects from the univariate analysis of variance (±SD) are presented; significance of effects: * = *p* < 0.05, ** = *p* < 0.01, *** = *p* < 0.001.

PV was primarily influenced by LBM (*ƞ* ^2^ = 0.44), with sex having only a marginal impact (*ƞ* ^2^ = 0.063). Altitude had a negative effect (*ƞ* ^2^ = 0.076), which became significant at an altitude of 1730 m and above (Table 4).

In addition to sex (*ƞ* ^2^ = 0.45) [Hb] was influenced by altitude (*ƞ* ^2^ = 0.22), which was significant in residents at 1730 m and higher, but not by LBM. There was a significant relationship between the interaction of LBM and sex for Hbmass, but not for LBM and sex for PV. There were also no interactions between sex and altitude for Hbmass, PV, or [Hb] (*p* = 0.49, *p* = 0.93, and *p* = 0.18, respectively).

Multiple regression analysis shows that Hbmass and PV are both strongly related to [Hb] (Table 5a). The separate effects of both variables are shown in Figure 2a,b. Further regression analysis was performed on altitude‐related differences using the altitude effects presented in Table 4, with Δ[Hb] as the dependent variable and ΔHbmass and ΔPV as the independent variables. The analysis revealed a significant influence for both variables, which were nearly equal in magnitude (Table 5b). The contribution to the 1.2 g/dL increase in [Hb] in residents at 2600 m was 0.56 g/dL for Hbmass and 0.61 g/dL for PV. However, when these analyses were performed with ΔPV_cf‐Sanchez_, the effect of PV far exceeded that of Hbmass (see Tables S2 and S3 in the Supplemental Material).

**TABLE 5:** Effect of sex, hemoglobin mass (Hbmass), plasma volume (PV) and peripheral O_2_‐saturation (SpO_2_) on hemoglobin concentration ([Hb]).

A	Sex (male)	Hbmass (100 g)	PV (L)	SpO_2_ (%)	Constant term	*R* ^2^
[Hb] (g/dL)	0.30*** ±0.06	1.41*** ±0.03	−3.28*** ±0.07	0.04*** ±0.01	11.09*** ±1.17	0.954

B		ΔHbmass (100 g)	ΔPV (L)			
Δ[Hb] (g/dL)	__	1.59** ±0.28	−3.07** ±0.56	__	0.05 ±0.08	0.969

*Note*: Presented are the results of multiple regression analyses: A: regression coefficients (±SD) for the independent variables sex, Hbmass, PV, and SpO_2_ on [Hb]; B: regression coefficients for the independent variables “altitude effects of Hbmass and PV” obtained from Table 4 on the altitude related differences in [Hb]. Significance of effects: ** = *p* < 0.01, *** = *p* < 0.001.

**FIGURE 2:**
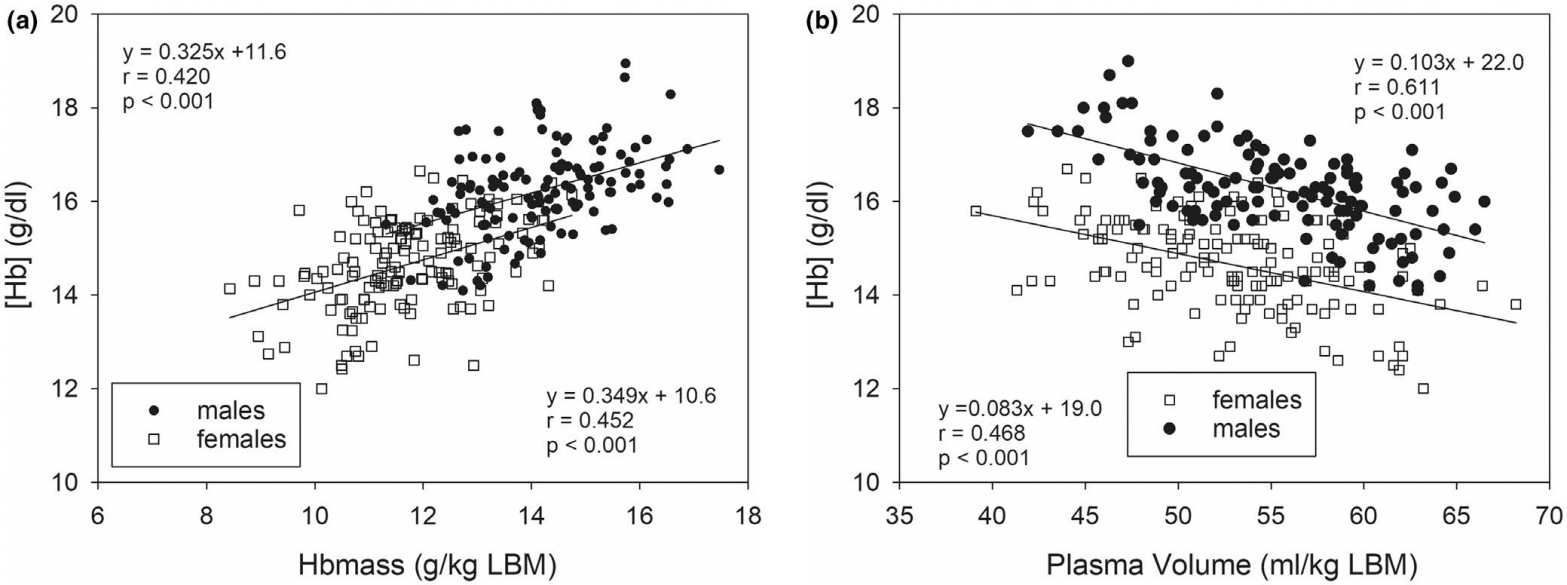
Relationship between (a) hemoglobin mass (Hbmass) and hemoglobin concentration ([Hb]) and (b) plasma volume (PV) and [Hb]. Data and statistical outcomes are separated for males (upper corners) and females (lower corners).

## 4 DISCUSSION

The most important finding of this study is the determination of altitude thresholds for significant increases in Hbmass and decreases in PV in Andeans. Residence greater than or equal to 2180 m led to higher Hbmass, whereas residence greater than or equal to 1730 m led to lower PV. The higher Hbmass and the lower PV contribute roughly equally to the increase in [Hb] at moderate altitudes.

### 4.1 Hemoglobin mass (Hbmass)

Hemoglobin mass at moderate altitudes has primarily been studied to monitor the effectiveness of erythropoiesis in altitude training. Studies on chronic adaptations have been almost exclusively selective, comparing high‐altitude populations with lowland dwellers (Böning et al., 2004; Mancera‐Soto et al., 2022; Schmidt et al., 2002; Schmidt et al., 2022). Weil et al. (1968) were the only researchers to compare multiple altitudes. They found no difference in erythrocyte volume at 1600 m compared to sea level, but they observed a significant increase at 3100 m. This increase showed a strong correlation with arterial SO_2_. These findings are consistent with a recent review (Siebenmann et al., 2024), which reported that the minimum altitude for substantial red blood cell volume expansion is ~3000 m. In the present study, Hbmass was slightly but significantly elevated at 2180 m for the first time, though a tendency toward higher values was already present at 1920 m. A stable adaptation of Hbmass can be observed at an altitude of 2600 m, which is consistent with the results of our previous studies at the same altitude (Mancera‐Soto et al., 2022).

Notably, Hbmass values vary considerably among individuals at all altitudes. The mean standard deviation is 108 g for males and 65 g for females at their respective altitudes. This far exceeds the 36 g isolated altitude effect at 2600 meters (see Table 4). This difference is primarily due to variations in LBM levels among individuals as indicated by the high value for *ƞ* ^2^. However, Hbmass data related to LBM show similar standard deviations for both the male and female altitude groups (~1.1 g/kg LBM) compared to the maximum altitude effect (~1.0 g/kg LBM; see Table 3). Therefore, the effect of altitude on erythropoiesis at moderate altitudes is just one of several confounding individual factors. One of these factors could be the varying ambient temperatures at the six different altitudes (see Table 1). Passive heat acclimatization over a five‐week period increases Hbmass by approximately 4% (Jenkins et al., 2025). Therefore, it seems possible that high ambient temperatures at low altitudes partially offset the effect of altitude on erythropoiesis. However, when a relatively homogeneous population—such as elite athletes—is exposed to altitude, a significant erythropoietic effect is observed even at lower altitudes (1800 m) (Garvican‐Lewis et al., 2015) than in this comparison of the general population. In the present study, the influence of altitude is partly masked by a variety of other factors.

Combining the Hbmass data from this study with that from our earlier study (Schmidt et al., 2022), which used the same methodology at 420 and 3800 m, shows a slow increase until 2600 m followed by a steep one (Figure 3a). Values are 27% higher at 3800 m than at near sea level, consistent with data from even higher altitudes (Oberholzer et al., 2020). This steep increase is due to the sigmoid shape of the ODC, which enables near‐optimal arterial oxygen saturation up to approximately 2000 m, after which it drops sharply (Schmidt, Dahners, et al., 1990). As demonstrated in Figure 3b, there is a significant relationship between Hbmass and SpO_2_ when the data from both studies are combined. However, when only the data for residents up to 2600 meters are considered, no significant relationship is found. This suggests that, up to an altitude of about 2600 meters, altitude only slightly affects erythropoiesis. Above that altitude, the lower amount of available oxygen, most likely registered in the kidneys (Montero & Lundby, 2019), becomes more impactful (Montero & Lundby, 2019).

**FIGURE 3:**
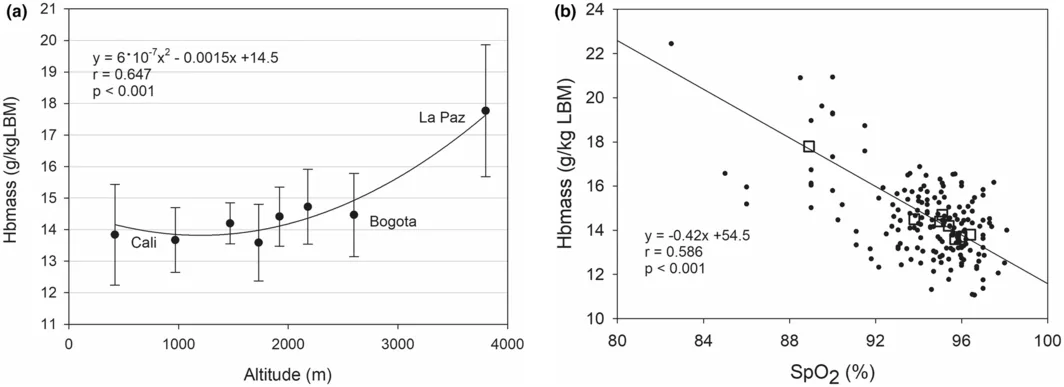
Hemoglobin mass (Hbmass) in residents of different altitudes. Presented are data from this study (altitudes between 970 and 2600 m) and from a former study of Bolivian residents (420 and 3800 m) published under the CC BY 4.0 license Schmidt, Wachsmuth, Jimenez and Soria, 2022. (a) Relationship between Hbmass (g/kg LBM, mean and SD) and altitude of residence. (b) Relationship between Hbmass and peripheral oxygen saturation (SpO_2_); open squares represent the mean values of the respective altitude.

The mechanism by which hemoglobin increases at higher altitudes is generally attributed to higher levels of erythropoietin (EPO). This occurs during short stays at high altitudes, when the EPO level peaks around the second day (Heinicke et al., 2003). However, it subsequently drops significantly. Among residents of moderate altitudes (2600–3600 m), no difference can be detected compared to residents of lower altitudes, despite significantly higher Hbmass levels (Heinicke et al., 2003; Mancera‐Soto et al., 2021). One possible explanation is that residents of high altitudes have a higher EPO receptor density, which binds more EPO and increases erythropoietic activity without affecting the plasma EPO concentration (Villafuerte et al., 2014).

### 4.2 Plasma volume (PV)

It is well known that PV changes with altitude; that is, it decreases during ascent and increases during descent. These changes occur within a few days, and their magnitude depends on the altitude difference. After ascending to 2500 meters, PV is approximately 6% lower after 1 day and 13% lower after 7 days (Beidleman et al., 2017). Conversely, when high‐altitude residents from 2600 m descend to an altitude of approximately 400 m, their PV is measured to be 13% higher after 1 day and 17% higher after 7 days (Cristancho et al., 2026). A comparison of moderate‐altitude and sea‐level residents shows a similar difference in PV as seen after acute ascent (Böning et al., 2004; Mancera‐Soto et al., 2022), thus proving chronic adaptation. However, it is not yet known at what altitude PV reduction occurs when compared to sea level.

One problem with answering this question is that PV is rarely measured directly. The method involving Evans Blue and radioactive tracers that was used several decades ago is no longer feasible. Therefore, PV is now determined indirectly by measuring Hbmass or red cell volume, and then calculating PV based on hematocrit. Using this method, the Fåhraeus effect is thought to affect the total amount of blood in the body by about 9%, but this may not be true at higher altitudes. Regardless of hematocrit, Sanchez et al. (1970) found an 11% difference between total blood volume and peripheral volume at 4450 m, compared to 9% at sea level, through separate measurements of RCV and PV. Therefore, we applied an additional correction according to Sánchez, which increased the magnitude of the PV reduction at higher altitudes (see Table 1, Supplementary Material) compared to the conventional calculation. However, this correction had a minor impact on the statistical conclusions. That is, compared to the Hbmass threshold, the PV threshold also seems to be ~450 m lower with this calculation (Table 2, Supplementary Material).

There is little data in the literature on the mechanism of volume contraction in residents of moderate altitudes. As with acute altitude exposure, it is possible that there is adapted hormonal regulation of the electrolyte and fluid balance. This mechanism involves moderate suppression of the renin‐angiotensin‐aldosterone system, as well as activation of ANP, which increases diuresis (Siebenmann et al., 2015). However, recent studies (Siebenmann et al., 2024) indicate that, particularly during prolonged stays at high altitudes, there is a redistribution of water from the intravascular to the extravascular compartment rather than altitude diuresis. It is unclear whether this applies to chronic exposure to high altitudes or if it is the only or main change in fluid regulation compared to sea level.

The biological reason for an acute decrease in PV during ascent is to adapt the body's oxygen‐carrying capacity to an oxygen deficit. The reason for chronically reduced PV, however, may be to prevent hypervolemia. This is achieved at the moderate altitudes studied here. However, at very high altitudes, the increase in RCV exceeds the decrease in PV, so hypervolemia cannot be prevented (Hurtado, 1960; Oberholzer et al., 2020; Schmidt et al., 2022).

Another reason for the higher PV at lower altitudes could be the higher ambient temperatures found there (see Table 1). Under moderate climatic conditions with seasonal variations, PV is known to be approximately 8% higher in the summer than in the winter (Sawka et al., 2000). Therefore, it cannot be ruled out that the differences in PV are at least partly due to climatic factors rather than adaptation to altitude.

### 4.3 Hemoglobin concentration ([Hb])

The altitude‐related increase in [Hb] in this study is consistent with the literature. In their meta‐analyses, Gassmann et al. (2019) and Mairbäurl et al. (2023) describe greater increases in [Hb] in Andean residents than in high‐altitude residents from East Africa and the Himalayan region. The higher values at higher altitudes, which are significant from 1730 m and amount to 1.2 g/dL at 2600 m (see Table 4), align well with the results of the linear regression equations for Andean residents reported by the aforementioned groups of authors.

Until now, the higher [Hb] levels associated with chronic high‐altitude residence have been primarily explained by increased erythropoiesis and the resulting greater Hbmass. However, recent data suggest that a reduction in PV also contributes to increased [Hb] levels, particularly at high altitudes (Oberholzer et al., 2020; Schmidt et al., 2022). The different [Hb] levels of high‐altitude dwellers in the Andes and the Himalayas also demonstrate such a mechanism. These differences in [Hb] are caused less by differences in Hbmass and more by differences in PV (Claydon et al., 2005; Stembridge et al., 2019; Tremblay et al., 2025). In this study, we are able to quantify the impact of changes in Hbmass and PV on increased [Hb] at moderate altitudes. Our calculations show that a 100 g increase in Hbmass increases [Hb] by 1.59 g/dL and a 1 L decrease in PV increases [Hb] by 3.07 g/dL (see Table 5b). These estimates demonstrate that the 1.2 g/dL elevated [Hb] at 2600 meters is equally due to higher Hbmass and lower PV.

The question arises whether these findings have practical significance. The [Hb] value, which defines the threshold for anemia, increases with altitude (Gassmann et al., 2019; Mairbäurl et al., 2023). Therefore, it should be considered whether hemoconcentration occurring at high altitudes should be taken into account in such definitions, which has not yet been done.

## 5 LIMITATIONS

The main limitation of this study is the relatively small number of participants at each altitude. Although this is, to our knowledge, the first study to determine Hbmass and PV at different altitudes and includes 281 individuals, the high interindividual variability requires a larger sample size to draw general conclusions. Additionally, we can only draw conclusions for the region we studied. Other high‐altitude populations, such as those in Tibet and East Africa, exhibit different adaptations to altitude.

Quantitative statements about PV should be treated with caution because they are based on calculations in which the Fåhraeus effects can only be estimated. Therefore, in addition to the conventional calculation, we performed an altitude correction. This correction amplifies the impact of altitude on PV, thereby confirming our conclusions.

We used an impedance method to determine LBM, which is less accurate than the DEXA method. However, since all of our subjects were in the low BMI range, any potential errors can be considered minor. This is also demonstrated by the finding that the effects of altitude on Hbmass and PV, as well as their impact on [Hb], are valid when analyses are performed using LBM‐based values.

## 6 CONCLUSIONS

The altitude threshold for chronic erythropoietic adaptation in Andeans was determined to be 2180 m. By contrast, the threshold for a reduction in PV was approximately 450 m lower. For the population studied at moderate altitudes, a reduction in PV contributes as much as an increase in Hbmass to the increase in [Hb].

## AUTHOR CONTRIBUTIONS

**Edgar Cristancho‐Mejía:** Conceptualization; data curation; formal analysis; funding acquisition; investigation; methodology; project administration; resources; supervision; validation. **Andrea Catalina Trompetero‐Gonzalez:** Data curation; investigation; methodology; software; validation. **William Fernando Benavides‐Pinzón:** Data curation; investigation; methodology; software; validation. **Walter Franz‐Joachim Schmidt:** Conceptualization; data curation; formal analysis; investigation; methodology; project administration; resources; software; supervision; validation; visualization.

## FUNDING INFORMATION

The study was funded by regular grants (Grant number 110152128674) of Fondo Colombiano de Investigaciones Científicas y Proyectos Especiales “Francisco José de Caldas,” Colciencias.

## CONFLICT OF INTEREST STATEMENT

Professor Schmidt is a co‐owner of Blood tec GmbH and receives a portion of its profits. However, this study has no grants or other financial dependencies related to Blood tec GmbH. The remaining authors declare that there is no conflict of interest.

## ETHICS STATEMENT

The Ethics Committee of the Faculty of Natural Sciences at the National University of Colombia in Bogotá approved all procedures (April 24, 2009) and the experiments were conducted in accordance with the ethical standards outlined in the 1964 Declaration of Helsinki. Written informed consent was obtained from all individual participants included in the study.

## Supporting information


Data S1.

## Data Availability

The data that support this study's findings are available from the corresponding author upon reasonable request.
