# Supplementary material for: Altitude thresholds for hemoglobin and plasma volume regulation in adult residents of different altitudes and their impact on hemoglobin concentration
Source: Physiol Rep. 2026 Oct 2;14(19):e71108. doi: 10.14814/phy2.71108 (PMC13633473; doi:10.14814/phy2.71108)
Supplement: Supplementary file 1 [file PHY2-14-e71108-s001.docx]

Table 1: Plasma and blood volumes calculated using the cell factor (cf_Sanchez_)

| Males | PV  ml | BV  ml | PV  g/kg | BV  g/kg | PV  ml/kgLBM | BV  ml/kgLBM |
| --- | --- | --- | --- | --- | --- | --- |
| 970m  n=17 | 3501  ±686 | 5845  ±1110 | 50.0  ±4.6 | 83.5  ±6.1 | 57.8  ±5.8 | 96.5  ±7.9 |
| 1470m  n=12 | 3216  ±538 | 5487  ±788 | 51.0  ±4.3 | 87.1  ±5.8 | 56.3  ±4.6 | 96.8  ±4.8 |
| 1728m  n=22 | 3094**  ±495 | 5299*  ±799 | 47.2  ±7.0 | 80.8  ±10.9 | 54.5  ±5.6 | 93.2  ±8.2 |
| 1923m  n=15 | 2763***  ±322 | 4906***  ±552 | 47.3  ±4.7 | 84.0  ±7.1 | 52.9*  ±5.2 | 93.9  ±7.3 |
| 2180m  n=12 | 2989**  ±400 | 5274**  ±609 | 47.7  ±4.0 | 84.4  ±5.1 | 54.9  ±4.9 | 97.0  ±6.1 |
| 2600m  n=55 | 2835**  ±351 | 5135***  ±566 | 45.0**  ±4.3 | 81.4  ±7.8 | 51.5***  ±5.6 | 93.2  ±8.0 |
| ANOVA  P < | 0.001 | 0.01 | 0.01 |  | 0.001 |  |
| Females |  |  |  |  |  |  |
| 970m  n=28 | 2408  ±242 | 3820  ±404 | 43.8  ±4.1 | 69.4  ±6.0 | 55.9  ±4.6 | 88.6  ±6.8 |
| 1470m  n=11 | 2384  ±281 | 3896  ±463 | 42.1  ±6.2 | 68.8  ±10.1 | 54.1  ±7.6 | 88.6  ±12.7 |
| 1728m  n=23 | 2185**  ±375 | 3546*  ±515 | 41.7  ±6.7 | 67.7  ±8.5 | 52.4*  ±7.8 | 85.0  ±10.4 |
| 1923m  n=18 | 1981***  ±244 | 3378**  ±443 | 38.9**  ±5.1 | 66.1  ±8.1 | 48.7***  ±4.7 | 82.9  ±7.8 |
| 2180m  n=21 | 2218*  ±338 | 3735  ±514 | 39.1**  ±6.2 | 65.7  ±8.4 | 51.2**  ±6.6 | 86.1  ±8.7 |
| 2600m  n=48 | 2147***  ±263 | 3399  ±408 | 38.6***  ±4.8 | 66.4  ±7.0 | 49.9***  ±5.2 | 86.0  ±7.8 |
| ANOVA  P < | 0.001 | 0.05 | 0.01 |  | 0.001 |  |

(cf_Sanchez_) = Cell factor calculated according to Sanchez C, Merino C, Figallo M. Simultaneous measurement of plasma volume and cell mass in polycythemia of high altitude. J Appl Physiol. 1970;28(6):775-778.

Significance of effects: * = p< 0.05, ** = p< 0.01, *** = p<0.001.

Table 2: Effect of sex, altitude, and lean body mass (LBM) on plasma volume calculated according to Sanchez (PV_Sanchez_)

|  | sex | altitude | | | | | LBM | constant  term |
| --- | --- | --- | --- | --- | --- | --- | --- | --- |
|  | male | 1470m | 1730m | 1920m | 2170m | 2600m | kg |  |
| PV_Sanchez_  (ml) | 212**  ±55 | -119  ±78 | -190**  ±61 | -339***  ±68 | -227***  ±64 | -343***  ±53 | 46.1***  ±3.3 | 665***  ±197 |

Significance of effects: * = p< 0.05, ** = p< 0.01, *** = p<0.001.

Table 3: Effect of sex, hemoglobin mass (Hbmass), plasma volume calculated according to Sanchez (PV_Sanchez_) and peripheral O_2_-saturation (SpO_2_) on hemoglobin concentration ([Hb]); A. absolute values, B: differences from 970 m

| **A** | sex  (male) | Hbmass  (100 g) | PV  (l) | SpO_2_  (%) | constant term | R^2^ |
| --- | --- | --- | --- | --- | --- | --- |
| [Hb]  (g/dl) | 0.33***  ±0.07 | 1.28***  ±0.03 | -3.07***  ±0.06 | 0.08***  ±0.01 | 6.57***  ±1.30 | 0.944 |
|  |  |  |  |  |  |  |
| **B** |  | ΔHbmass (100 g) | ΔPV  (l) |  |  |  |
| Δ[Hb]  (g/dl) | __ | 1.08*  ±0.29 | -2.46**  ±0.40 | __ | 0.04  ±0.08 | 0.976 |

Significance of effects: * = p< 0.05, ** = p< 0.01, *** = p<0.001.
